# Cutaneous Distribution and Circadian Rhythm of *Onchocerca lupi* Microfilariae in Dogs

**DOI:** 10.1371/journal.pntd.0002585

**Published:** 2013-12-12

**Authors:** Domenico Otranto, Filipe Dantas-Torres, Alessio Giannelli, Francesca Abramo, Aleksandra Ignjatović Ćupina, Dušan Petrić, Luís Cardoso, Yasen Mutafchiev, Helder Cortes

**Affiliations:** 1 Department of Veterinary Medicine, University of Bari, Valenzano, Bari, Italy; 2 Department of Immunology, Aggeu Magalãhes Research Institute, Oswaldo Cruz Foundation, Recife, Pernambuco, Brazil; 3 Department of Animal Pathology, University of Pisa, Pisa, Italy; 4 Faculty of Agriculture, University of Novi Sad, Novi Sad, Serbia; 5 Department of Veterinary Sciences, School of Agrarian and Veterinary Sciences, University of Trás-os-Montes e Alto Douro, Vila Real, and Instituto de Biologia Molecular e Celular, Universidade do Porto, Oporto, Portugal; 6 Institute of Biodiversity and Ecosystem Research, Bulgarian Academy of Sciences, Sofia, Bulgaria; 7 Instituto de Ciências Agrárias e Ambientais Mediterrânicas, Universidade de Évora, Núcleo da Mitra, Évora, Portugal; Hebrew University, Israel

## Abstract

**Background:**

Among the arthropod-borne nematodes infesting dogs, *Onchocerca lupi* (Spirurida: Onchocercidae) is of increasing zoonotic concern, with new human cases of infection diagnosed in Turkey, Tunisia, Iran and the USA. Knowledge of the biology of this nematode is meagre. This study aimed at assessing the distribution and periodicity of *O. lupi* microfilariae from different body regions in naturally infested dogs.

**Methodology/Principal Findings:**

Skin samples were collected from six dogs infested with *O. lupi* but without apparent clinical signs. Two skin samples were collected from 18 anatomical regions of dog 1 at necropsy. In addition, single skin biopsies were performed from the forehead, inter-scapular and lumbar regions of dogs 2–6, in the morning, afternoon, and at night. Two aliquots of the sediment of each sample were microscopically observed, microfilariae counted and morphologically and molecularly identified. Most of the 1,667 microfilariae retrieved from dog 1 were in the right ear (59.6%), nose (26.5%), left ear (6.7%), forehead (3.0%), and inter-scapular (2.9%) regions. In dogs 2–6, the overall mean number of microfilariae was larger on the head (n = 122.8), followed by the inter-scapular (n = 119.0) and lumbar (n = 12.8) regions. The overall mean number of microfilariae was larger in the afternoon (153.4), followed by night (75.4) and morning (25.8).

**Conclusions:**

*Onchocerca lupi* microfilariae were more common in the head (i.e., ears and nose) than in the remaining part of the dog's body, indicating they tend to aggregate in specific body regions, which are the best sites to collect skin samples for diagnostic purposes. The periodicity pattern of microfilariae of *O. lupi* and their concentration in specific body regions is most likely a result of the co-evolution with their as-yet-unknown vector. The detection of skin microfilariae in asymptomatic animals, suggests the potential role of these animals as carriers and reservoirs of *O. lupi*.

## Author Summary

*Onchocerca lupi* is a little known arthropod-borne helminth infesting dogs of increasing interest to the scientific community due to the recent demonstration of its zoonotic potential. Nonetheless, knowledge of the biology of this nematode is exiguous. In this study the distribution and periodicity of *O. lupi* microfilariae was investigated from different body regions in naturally infested dogs. Data indicate that *O. lupi* microfilariae were more common in the head (i.e., ears and nose) followed by the inter-scapular region than in the remaining part of the dog's body suggesting that these parasites aggregate in these anatomical sites. These regions might be the best sites to collect skin samples for diagnostic purposes. Finally, the periodicity pattern of microfilariae of *O. lupi* and their concentration in specific body regions is most likely a result of the co-evolution with their as-yet-unknown vector.

## Introduction

Vector-borne nematodes of the family Onchocercidae (Spirurida) are of major medical concern. Among others, adult worms of *Wuchereria bancrofti* and *Brugia malayi* may live in the lymphatic system of humans causing obstruction (i.e., elephantiasis) and those of *Onchocerca volvulus* in the subcutaneous tissues, with microfilariae inducing systemic or localized abnormal immune-mediated response, ultimately leading to severe ocular onchocercosis [1]. Some of these diseases may impact human health; for instance, the so-called “river blindness” caused by *O. volvulus* affects about 17.7 million people globally [2]. Among the arthropod-borne helminths of dogs, an increasing zoonotic role is recognized for *Dirofilaria immitis* and *Dirofilaria repens*, which are characterized by blood circulating microfilariae that may eventually infest the eyes and other organs of patients [3]. In contrast, data on the biology of onchocercid nematodes of the genera *Onchocerca* and *Cercopithifilaria*, characterized by subcutaneous localized microfilariae in dogs, is meagre [3]. *Onchocerca lupi*, a parasite of the connective tissue of sclera, has been sporadically reported in symptomatic dogs from Hungary, Greece, Germany and Portugal [4]–[7] and, more recently, also in dogs and cats from the USA [8], [9]. In dogs, this filarial worm may cause ocular lesions ranging from no apparent clinical sings [10] to blindness [11], with subconjunctival granulomas representing the finding most commonly reported [5]. A recent study on 107 dogs sampled in Greece and Portugal reveals an overall prevalence of infestation with *O. lupi* of 8.4% [10].

Since the first report of human ocular infestation by *O. lupi* [12] this parasite has been recognised as a zoonotic agent in patients from Turkey, Tunisia [13], Iran [14] and the USA [15]. Despite the resurrected interest of scientific community towards this onchocercid, knowledge of its biology remains obscure and its vectors are still unknown. Therefore, this study aimed at assessing the distribution, abundance and periodicity of *O. lupi* microfilariae collected from different body regions in six naturally infested dogs.

## Materials and Methods

### Sample collection

The study was conducted according to the principles of Good Clinical Practice (VICH GL9 GCP, 2000 http://www.ema.europa.eu/docs/en_GB/document_library/Scientific_guideline/2009/10/WC500004343.pdf) and procedures were approved by the Ethical commission at the University of Évora (identification number: AE02Fila2013) as complying with the Portuguese legislation for the protection of animals (Law no. 92/1995, from 12th of September). An owner consent agreement was obtained before sampling collection.

### Study design and experimental procedures

On March 2013, skin samples were collected from six mongrel dogs (i.e., two males and four females), from four to 10 years of age, living in the municipality of Olhão, Algarve region, southern Portugal (latitude 37°01′42″N, longitude 7°50′33″W, 8 meters above the sea level). All animals were previously identified as infested with *O. lupi* by the examination of skin snip sediments, during an epidemiological survey conducted in the study area [10]; none of the dogs had received endo- or ecto-parasitic treatments.

One of the dogs (dog 1) accidentally died due to gastric volvulus and, during necropsy, two skin samples were collected from 18 anatomical regions (about 2 cm apart), distributed throughout the body surface (Table 1). In addition, single skin biopsies were performed from the remaining five dogs (i.e., dog 2–6), from three anatomical regions (i.e., forehead, inter-scapular and lumbar regions) at different time points (i.e., in the morning at 10:00, late afternoon at 18:00, and during the night at 23:00 h).

**Table 1:** Number of *O. lupi* microfilariae retrieved by soaking skin samples from each anatomical site collected at the necropsy of dog 1.

Anatomical sites	Replicate 1 (after 6 h)	Replicate 2 (after 12 h)	Total/body region
Nose	122	319	441
Forehead	31	19	50
Left ear	10	102	112
Right ear	86	908	994
Inter-scapular	3	46	49
Low back	1	2	3
Left forelimb	0	2	2
Right forelimb	0	0	0
Left armpit	0	0	0
Right armpit	0	0	0
Left back limb	3	2	5
Right back limb	1	0	1
Left inguinal	0	0	0
Right inguinal	0	0	0
Abdomen	0	0	0
Back	2	3	5
Thorax	0	4	4
Neck	1	0	1
Total	260	1,407	1,667

Larvae were counted after 6 h and 12 h of soaking (i.e. replicate 1 and 2) by reading two aliquots (each by 20 µl) of sediment.

All skin samples from the six dogs were collected using biopsy punches (4 mm in diameter) and soaked in 2 ml saline solution (NaCl 0.9%) before observation.

### Diagnostic procedures

**Figure 1:**
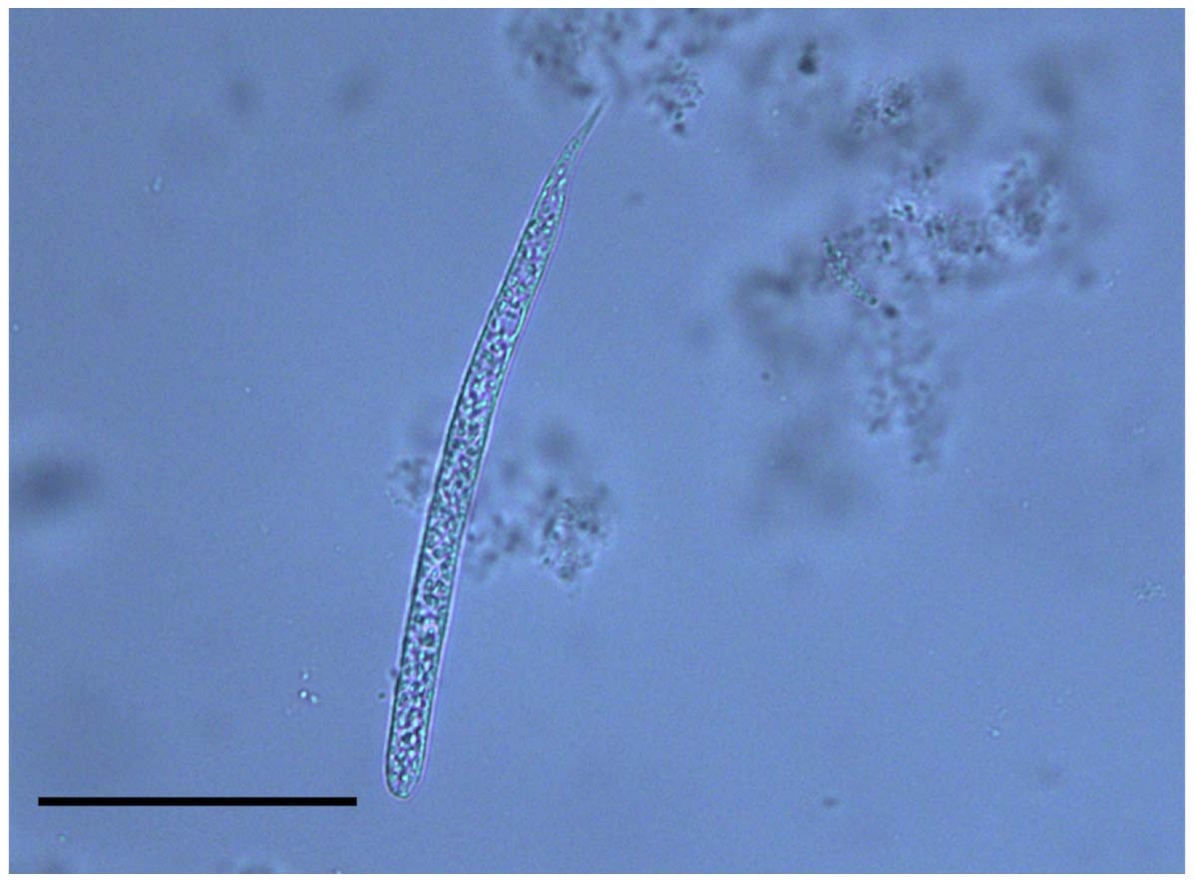
Microfilaria of *Onchocerca lupi*. Microfilaria of *Onchocerca lupi* found in the skin sediment of a dog (Scale bar: 50 µm).

For each sample, two aliquots (20 µl each) of the sediment were used to prepare temporary mounts, covered by an 18×18 mm coverslip, which were observed under a light microscope. Microfilariae were identified according to their morphology [11], [16]. Briefly, *O. lupi* microfilariae are characterised by an unsheathed body 110.1 µm ± 7.5 SD long and 6.8 µm ± 1.2 SD wide, rounded anterior extremity bearing a tiny tooth and a bent tail 11.7 µm long Figure 1. Additionally, three biopsy punches (8 mm in diameter) were taken from the nose and the peri-ocular regions at the necropsy of dog 1 for histological examination (see below). These skin samples were fixed in 4% buffered formalin solution (pH 7.4), embedded in paraffin and routinely processed for light microscopy. Thick sections (5 µm) were stained with haematoxylin and eosin before being microscopically observed.

The morphological identification was confirmed by molecular amplification and sequencing of the partial cytochrome oxidase subunit 1 (*cox*1) gene, following procedures described elsewhere [13]. Nucleotide sequences, examined by BLAST tool, displayed 100% homology with sequences of *O. lupi* from Portugal deposited in GenBank (accession number: EF521410).

Skin samples from dog 1 were soaked in saline solution for approximately 6 h (first replicate) and 12 h (second replicate) before observation, whereas samples from dog 2–6 were counted in a single assessment within 12 h after collection.

### Statistical analysis

The mean number (± standard deviation) of microfilariae was calculated according to body location and periodicity. Data normality was assessed using Lilliefors test and then the mean number of microfilariae according to collection site and period was compared using one way ANOVA, with Tukey post hoc test or Mann-Whitney U test as appropriate. A p<0.05 was considered statistically significant. Statistical analysis was conducted using BioEstat (version 5.0; Mamiraua/CNPq, Belem, PA, Brazil).

## Results

**Figure 2:**
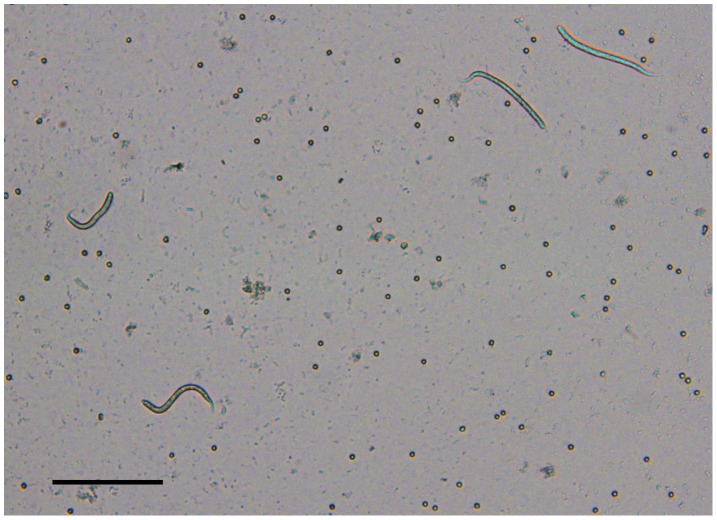
*Onchocerca lupi* microfilariae in the skin sediment. Several *Onchocerca lupi* microfilariae collected from the right ear of dog 1 (second replicate) (Scale bar: 100 µm).

All sampled animals were apparently healthy, presenting no apparent ocular alteration. The number of *O. lupi* microfilariae from each body site assessed at the necropsy of dog 1 is reported in Table 1. A total of 1,667 microfilariae of *O. lupi* were collected, most (95.8%) of which from the head. In particular, most of the microfilariae were located in the right ear (59.6%), nose (26.5%), left ear (6.7%), forehead (3.0%), and inter-scapular (2.9%) regions. Only 21 microfilariae (1.3%) were found in the remaining regions of the dog's body. Of the 12 body regions that resulted positive for microfilariae, eight were positive at both replicates (Table 1), with a higher percentage of skin samples positive at the examination of the first aliquot (n = 20; 71.4%) than of the second (n = 8; 28.6%; data not shown). Accordingly, the mean number of microfilariae counted in the first aliquot was higher than in the second (Mann-Whitney U test, p = 0.02), with up to 825 *O. lupi* microfilariae counted in a single sample from the right ear of dog 1 (Figure 2). The overall number of microfilariae retrieved in samples after 12 h of soaking (second replicate) was over 5 times higher than that after 6 h of soaking (first replicate). However, no significant difference was found in relation to the mean number of microfilariae/µl counted in each body site in the first and second replicates (Mann-Whitney U test, p = 0.37). Microfilariae were alive at both assessments.

**Figure 3:**
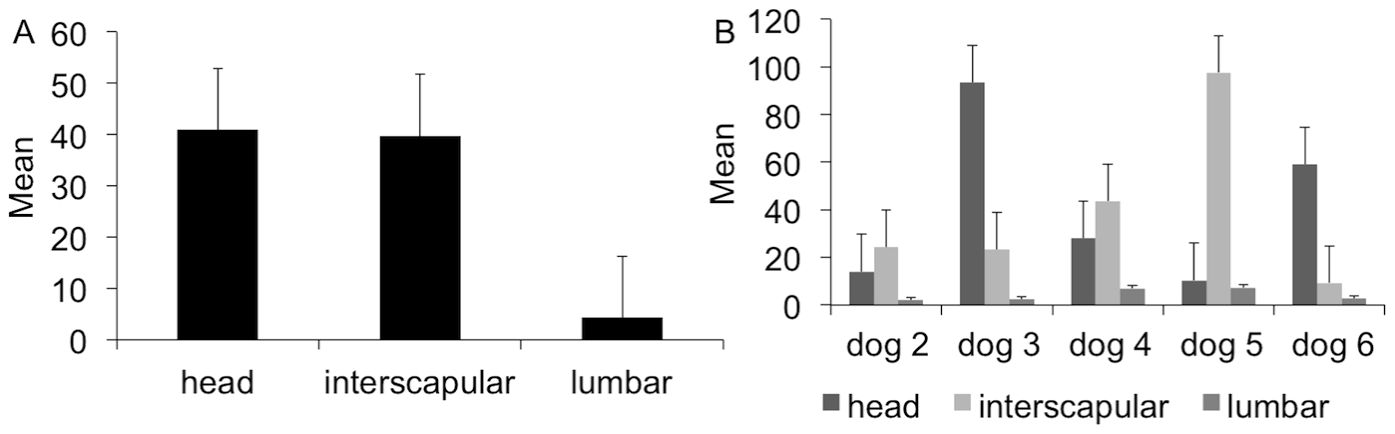
Distribution of microfilariae of *Onchocerca lupi*. Overall (A) and individual (B) mean number of microfilariae of *Onchocerca lupi* found in each body site of dogs 2–6.

In dogs 2–6, the mean number of microfilariae was higher on the head (40.9±35.0), followed by inter-scapular (39.7±34.6) and lumbar (4.3±2.7) regions (Figure 3a); however, no statistically significant difference was found in relation to body site (ANOVA, p = 0.11). The mean number of microfilariae per body site varied among dogs 2–6, with some dogs presenting more microfilariae in the head and others in the inter-scapular region (Figure 3b).

**Figure 4:**
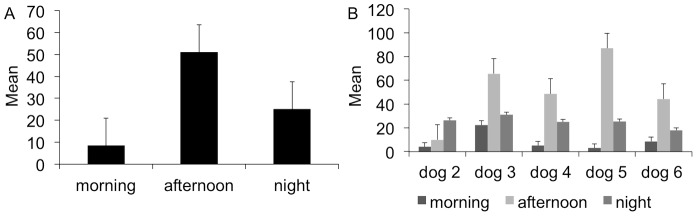
Circadian rhythm of *Onchocerca lupi* microfilariae. Overall (A) and individual (B) mean number of microfilariae of *Onchocerca lupi* retrieved from dogs 2–6, in the morning, late afternoon and at night.

As far as periodicity, the mean number of microfilariae was larger in the late afternoon (51.1±28.5), followed by night (25.1±4.7) and morning (8.6±8.0) (Figure 4a). Indeed, the mean number of microfilariae found in the morning sampling was significantly lower than that found in the late afternoon (ANOVA, p<0.01; Tukey post hoc test, p<0.01). Interestingly, the peak of microfilariae occurred during the night in dog 2 (Figure 4b).

**Figure 5:**
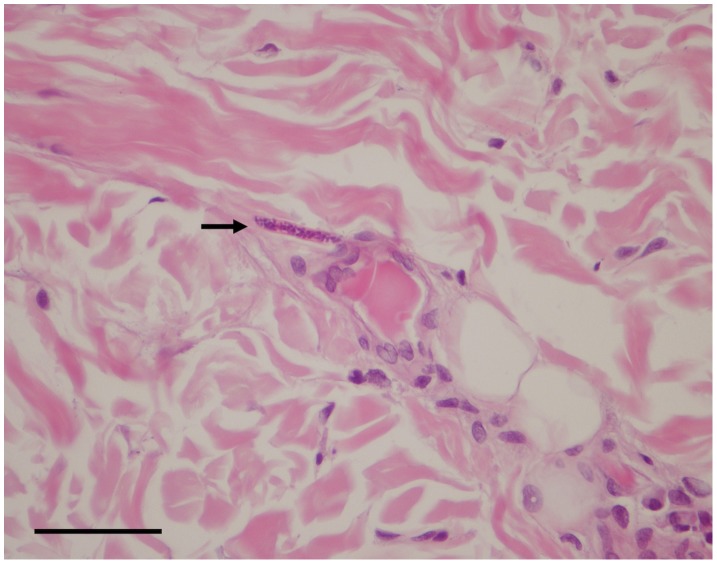
Histology of microfilaria of *Onchocerca lupi* in the skin. Microfilaria of *Onchocerca lupi* (arrow) detected in biopsied skin. In the interstitium of the dermis. Haematoxylin-eosin stain. Scale bar = 50 µm.

A few slender microfilariae were detected on histopathological examination of the peri-ocular regions in the dermis. They were unevenly distributed into the connective tissue among fibres in the perifollicular and interfollicular areas and in the deep dermis in the proximity of small vessels (Figure 5). Skin samples showed dermatitis with mild superficial and periadnexal perivascular infiltrates composed of eosinophils and a few lymphocytes. Inflammatory changes were accompanied by hyperplasia and ortokeratotic hyperkeratosis with a few coccoid bacteria between corneocytes.

## Discussion

Until now, information on the distribution and abundance of *O. lupi* microfilariae in the skin of infected dogs was limited to a single report on four symptomatic dogs sampled at the periocular and umbilical areas [17]. Interestingly, in spite of the absence of apparent clinical signs, animals were positive for skin microfilariae, suggesting the potential role of these animals as asymptomatic carriers and reservoirs of *O. lupi*.

Data on the distribution of *O. lupi* microfilariae (dog 1) showed they are more abundant in the head (i.e., ears and nose) than in the remaining part of the dog's body. Although this pattern was confirmed by the data on larval periodicity (dogs 2–6), in the latter case, the difference between the mean number of microfilariae from forehead and inter-scapular regions was not significant. This might be due to the fact that in dogs 2–6, skin was sampled from the forehead area, which, in turn, displayed a smaller number of microfilariae in comparison with ears and nose (Table 1). Consequently, the diagnosis of *O. lupi* infestation in dogs should be performed via the examination of skin samples collected from the ears or nose. Nonetheless, the inter-scapular region might be a preferred site because some dogs (dogs 2, 4 and 5) actually presented more microfilariae in the inter-scapular region in comparison with the forehead. Furthermore, the inter-scapular region is less vascularized and better accepted by both animals and owners as a site to be biopsied. In addition, this site might be more practical to be sampled during large population surveys [10].

The results of this study contrast with previous data on the distribution of *O. lupi* microfilariae [17], in which only peri-ocular and umbilical regions were considered as preferential sites for skin snipping. Although some authors [17] quantified the larval concentration (i.e., 267.5 larvae per gram of skin), they did not report the exact amount of skin tissue sampled and, most likely, underestimated the number of microfilariae due to the short period of soaking they adopted (i.e., 1 h). Indeed, the increased number of microfilariae at the second assessment (12 h) suggests that the longer is the duration of soaking, the highest is the probability to find microfilariae in the skin sediment. From a diagnostic perspective, microfilariae of *O. lupi* should also be differentiated from those of other filarial nematodes (i.e., *Cercopithifilaria bainae*, *Cercopithifilaria grassii* and *Cercopithifilaria* sp. II *sensu* Otranto et al. (2011), which may be retrieved at the same time in the dermis of dogs [18].

As recorded for other species within the genus, it becomes evident that *O. lupi* microfilariae tend to aggregate in specific body regions (i.e., head and inter-scapular region). This might be determined by the proximity of gravid *O. lupi* females to the sampling sites, as indicated in previous reports [11]. Similarly, microfilariae of *Onchocerca gutturosa*, *Onchocerca ochengi*, and *Ochocerca dukei* of cattle are found on dorsal side, posterior declivous abdomen and navel, respectively, in the same regions where adults are found [19]. Nonetheless, the adult localization of *Onchocerca* spp. is not mandatorily related to the preferred area of microfilariae localization, because the latter might migrate far away from females, through the lymphatic system [20], [21]. This is the case of microfilariae of *Onchocerca tarsicola* parasitizing red deer (*Cervus elaphus*), which are concentrated mainly in the external ears, whereas adults are present in the radial-carpal and tibia-tarsal joint tendons [22]. The histological evidence of mild skin eosinophilic inflammation nearby the microfilariae might be also a non-specific finding, not necessarily associated with the presence of the *O. lupi* larvae. Indeed, eosinophilic inflammation is usually seen in allergic as well as in parasitic skin diseases, thus other causes for such a condition cannot be ruled out. In addition, the minimal inflammatory response to *O. lupi* microfilariae could also be due to the fact they were recently released from a gravid female, as suggested for microfilariae of *D. immitis* [23].

The concentration of microfilariae of *O. volvulus* on the hip, shoulders and lower parts of the body [24] coincide with the sites where its black fly vectors (e.g., *Simulium damnosum* sensu lato) preferentially feed [25]. Undoubtedly, the occurrence of *O. lupi* microfilariae in specific body regions is most likely a result of the co-evolution between competent vectors, hosts and the parasite. Indeed, circadian variations of microfilariae reported in filarial worms with blood circulating microfilariae (e.g., *D. immitis*, *D. repens*, *Loa loa*, and *Wuchereria bancrofti*) is considered to be an adaptation to the biting behaviour of the vectors, the circadian rhythms of the host and to variations in environmental temperature and humidity [26]. This pattern has also been demonstrated for filarial worms with subcutaneous microfilariae as those of *O. volvulus*, in which the maximal larval density overlaps the peak of activity (i.e., between 18:00 and 19:00) of its *Simulium* vector [27], [28]. In the case of *O. lupi*, in absence of any scientific evidence, the role of mosquitoes (e.g., *Culex pipiens pipiens*, *Anopheles* spp.), or of biting midges species as vectors cannot be ruled out [3]. However, blackflies, whose biting activity increases in late morning or early afternoon [25], remain a major candidate as a vector of *O. lupi*. For example, *Simulium reptans*, a species collected where *O. lupi* cases have been reported in dogs (i.e., Germany, Greece, Hungary, Portugal and Switzerland) [29] displays exophilic and exophagic behaviours, with the highest biting activity during the afternoon [30]. Although suspected, the vector role of *S. reptans* has never been ascertained [29].

The information on the distribution and periodicity pattern of microfilariae of *O. lupi* here reported is of relevance not only for the comprehension of its biology, but also for a more refined diagnosis of the infestation. Indeed, in absence of any other diagnostic tool the “skin snip” remains the only option to detect larval stages in the subcutaneous tissue of infested dogs. Therefore, veterinary practitioners should be aware about the best body sites and period of the day for performing skin biopsy, in order to achieve a more reliable diagnosis toward a better comprehension of this little known parasite of increasing veterinary and medical concern.
